# Psychological wellbeing in a resource-limited work environment: examining levels and determinants among health workers in rural Malawi

**DOI:** 10.1186/s12960-019-0416-y

**Published:** 2019-11-15

**Authors:** Julia Lohmann, Olzhas Shulenbayev, Danielle Wilhelm, Adamson S. Muula, Manuela De Allegri

**Affiliations:** 1 London School of Hygiene & Tropical Medicine, 15-17 Tavistock Place, London, WC1H 9SH United Kingdom; 2 Heidelberg Institute of Global Health, University Hospital and Medical Faculty, Heidelberg University, Im Neuenheimer Feld 130.3, 69120 Heidelberg, Germany; 3 Department of Public Health, College of Medicine, University of Malawi, Private Bag 360, Chichiri, Blantyre 3, Malawi

**Keywords:** Psychological wellbeing, Malawi, Health workers, Determinants

## Abstract

**Background:**

A competent, responsive, and productive health workforce is central to a well-performing health system capable of providing universal access to high-quality care. Ensuring health workers’ psychological wellbeing is critical to sustaining their availability and productivity. This is particularly true in heavily constrained health systems in low- and lower-middle-income countries. Research on the issue, however, is scarce. This study aimed to contribute to filling the gap in knowledge by investigating levels of and factors associated with psychological wellbeing of mid-level health workers in Malawi.

**Methods:**

The study relied on a cross-sectional sample of 174 health workers from 33 primary- and secondary-level health facilities in four districts of Malawi. Psychological wellbeing was measured using the WHO-5 Wellbeing Index. Data were analyzed using linear and logistic regression models.

**Results:**

Twenty-five percent of respondents had WHO-5 scores indicative of poor psychological wellbeing. Analyses of factors related to psychological wellbeing showed no association with sex, cadre, having dependents, supervision, perceived coworker support, satisfaction with the physical work environment, satisfaction with remuneration, and motivation; a positive association with respondents’ satisfaction with interpersonal relationships at work; and a negative association with having received professional training recently. Results were inconclusive in regard to personal relationship status, seniority and responsibility at the health facility, clinical knowledge, perceived competence, perceived supervisor support, satisfaction with job demands, health facility level, data collection year, and exposure to performance-based financing.

**Conclusions:**

The high proportion of health workers with poor wellbeing scores is concerning in light of the general health workforce shortage in Malawi and strong links between wellbeing and work performance. While more research is needed to draw conclusions and provide recommendations as to how to enhance wellbeing, our results underline the importance of considering this as a key concern for human resources for health.

## Background

A competent, responsive, and productive health workforce is one of the World Health Organization’s (WHO) six essential building blocks of a well-performing health system which is capable of providing access to high-quality care [1]. Adequate availability, distribution, qualification, resourcing, and motivation of health workers are key determinants of such a productive workforce. In addition, ensuring health workers’ physical health as well as psychological wellbeing is crucial to sustaining their availability and productivity over time [2]. The latter, which we define as a continuum from perfect wellbeing at one end to clinically relevant, severe mental illness incapacitating a person’s daily functioning at the other end, is particularly important considering that health workers have been identified as being at high risk of poor psychological wellbeing due to their specific work demands [3, 4].

In high-income countries (HIC) and at an international level, both the importance of keeping the workforce psychologically healthy and the related key role that enabling and supportive working conditions play in sustaining health have long been recognized [5–7]. Numerous studies on the psychological wellbeing of health workers corroborate the importance of the issue. For instance, a survey of over 60 000 nurses in 2006/2007 found burnout rates ranging from around 10% in the Netherlands and in Switzerland to between 20 and 40% in other European countries and in the United States of America, and up to 78% in Greece [8]. Across countries and clinical settings, similar occupational determinants of poor psychological wellbeing among health care personnel have been identified [9], including excessive workload, inter- and intra-professional conflict, adverse management styles and poor management support, lack of autonomy, shift work, and effort-reward unbalance. In terms of consequences, poor psychological wellbeing has been linked to low quality of care [8], patient safety issues [10], poor empathic ability [11], and absenteeism [12].

In low- and lower-middle-income countries (LLMIC), in contrast, occupational health and particularly psychological wellbeing of the health workforce are seldom present in both the applied discourse and the academic literature on human resources for health (HRH) [13]. Empirical research is particularly scarce for mid-level health workers (i.e., nurses, midwives, and other clinically trained but non-physician staff) working at the primary and secondary health care levels, i.e., the backbone of health service provision in most LLMIC. Only ten studies could be identified, eight of which from sub-Saharan Africa (Ghana, Kenya, Malawi, Uganda, Zambia, Zimbabwe) and two from Asia (Pakistan, Thailand) [14–23]. These studies indicate that poor psychological wellbeing of health workers in LLMIC is an issue of concern. For instance, 68% of maternal health staff in one district hospital in Malawi [23] and 62% of health workers in two rural hospitals in Zambia [20] showed burnout symptoms. Three studies have looked at psychological wellbeing from a more holistic and continuous perspective. Studies in Uganda [19] and Zimbabwe [22] found relatively high levels of psychological wellbeing on average (around 80% of the maximum score), whereas wellbeing levels were around 50% of the maximum in Pakistan [14]. Four studies have investigated potential determinants of burnout, with mixed results in regard to age, seniority, gender, and work environment [14, 18, 19, 23]. Two studies have looked at the relationship between burnout and work outcomes, with higher burnout scores associated with stronger turnover intentions in Ghana [15] and with poorer self-reported quality of care in Thailand [21].

The small available body of evidence therefore underlines that poor psychological wellbeing of health workers is a substantial issue of concern and likely negatively associated with work outcomes, compromising patient care in already heavily constrained health systems. Existing evidence, however, is still very limited in geographic scope, with most studies conducted only in a few health facilities or health districts, and in its narrow focus on clinically relevant states of burnout as measured with either the Maslach Burnout Inventory or a two-item measure developed by Mbindyo and colleagues [17], both of which are not validated in the settings. There is a particular lack of studies investigating factors associated with psychological wellbeing beyond basic demographic characteristics.

Beyond a few common work stressors that may apply to health workers worldwide (e.g., high workload, irregular hours, constant confrontation with human suffering, effort-reward imbalance), fundamental differences in work realities between HIC and LLMIC [24] likely limit the transferability of evidence generated in high-income settings. More LLMIC-specific research is therefore urgently needed to sensitize decision-makers to the issue and to inform the development of preventive and mitigating strategies. This study aims to contribute to filling this gap in knowledge by providing evidence of levels of psychological wellbeing and factors associated with it among mid-level cadres in rural Malawi.

### Conceptual framework

The study conceptualizes psychological wellbeing, abbreviated as PW in the following, in alignment with WHO’s definition of mental health as “a state of wellbeing in which every individual realizes his or her own potential, can cope with the normal stresses of life, can work productively and fruitfully, and is able to make a contribution to her or his community” [25]. Specifically, PW is conceptualized along a spectrum that ranges from perfect wellbeing at one end to clinically relevant, severe mental illness incapacitating a person’s daily functioning at the other end, rather than as just the absence of psychopathological symptoms of a severity requiring treatment. We make this explicit distinction as from an applied HRH perspective, any suboptimal state of wellbeing possibly associated with reduced work performance is of interest, including but not limited to clinical states of mental illness.

**Fig. 1:**
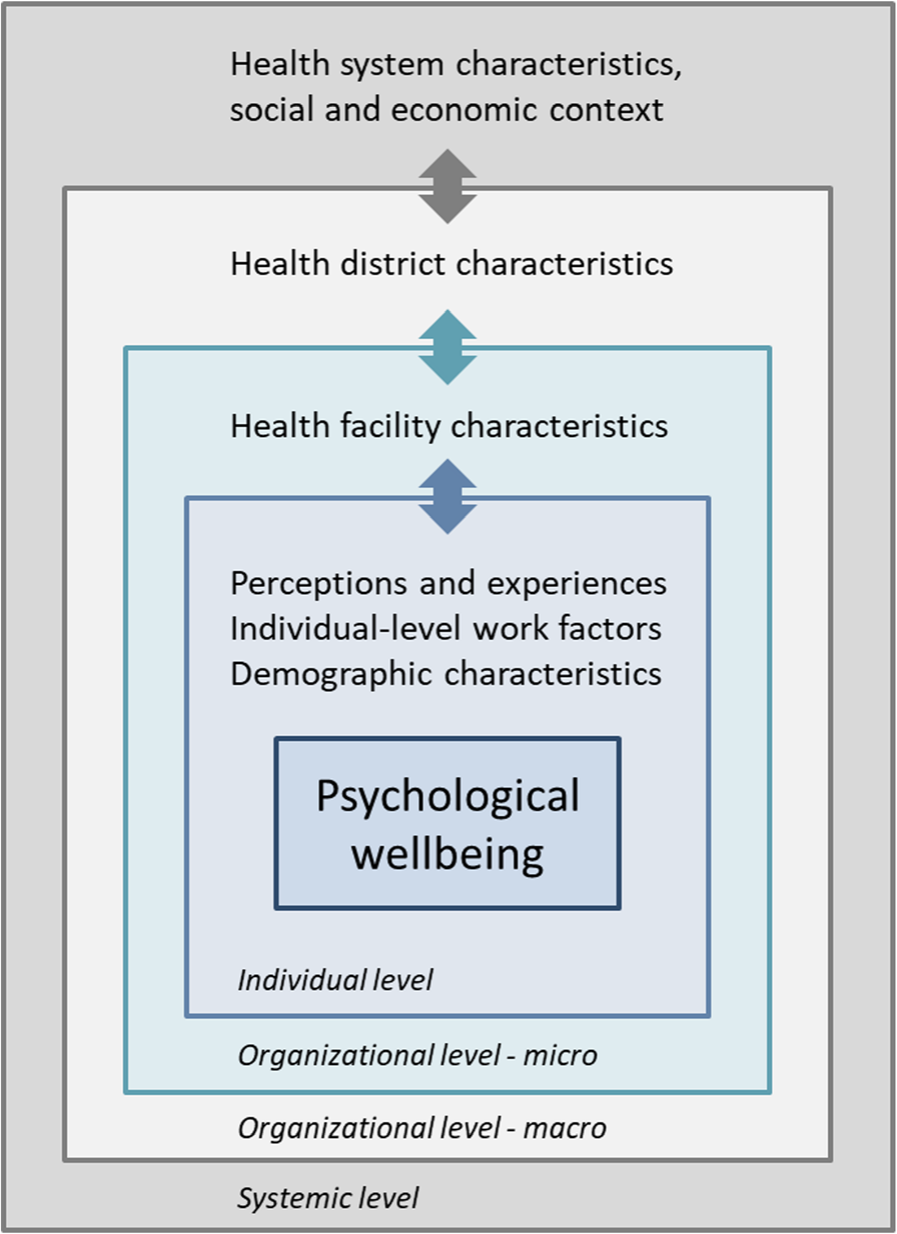
Conceptual framework

In line with the most commonly used taxonomy of determinants and consequences of occupational burnout [26] and based on the reviewed literature, the study further conceptualizes psychological wellbeing as embedded within a complex system of determinants and consequences at the individual, organizational, and broader systemic level (Fig. 1). At the individual level, it is assumed that in addition to demographic characteristics, diverse tangible individual-level work factors (e.g., cadre, training and knowledge, supervision) and intangible perceptions and experiences at work (e.g., satisfaction, motivation) directly affect health workers’ psychological wellbeing. These individual-level factors are assumed to be influenced by the organizational environment, including the physical work environment (e.g., availability of drugs, material, function equipment, adequate infrastructure), human resource availability and workload, the interpersonal work environment (e.g., service organization, team work), and managerial factors (e.g., leadership styles, managerial autonomy). The organizational environment, in turn, is assumed to be influenced by broader characteristics of the health system and the cultural, economic, and social context.

This study focuses on work-related individual-level factors associated with PW, specifically on factors potentially relevant to intervention design (e.g., key demographic characteristics) and on factors which can potentially be addressed by the health system (e.g., knowledge, satisfaction). The study explicitly does not address many work-unrelated factors associated with PW (e.g., personality), which, albeit important, are difficult to address through a health system intervention.

## Methods

### Context

The study took place in four rural health districts in Central and Southern Malawi, Balaka, Dedza, Ntcheu, and Mchinji. Despite substantial progress on various health indicators in recent years, the country continues to face a high mortality and morbidity burden due to communicable, non-communicable, and maternity-related conditions [27]. The Malawian health system is a predominantly public, government-funded three-tier system providing essential healthcare services to patients free of charge [28]. Health care service utilization is high [27], but provision of quality care is challenged by high workload levels due to severe health worker shortages, challenges in management and supervision, frequent stock-outs of drugs and other essential supplies, and other structural challenges [28–30]. Health workers are further frustrated with low salary levels and delays in payment thereof, limited and non-transparent career development opportunities, and lack of recognition of effort and good performance, as well as a variety of other factors [30, 31]. Despite working in difficult environments, Malawian health workers have expressed high levels of intrinsic motivation, pride in their work, and feelings of duty and of importance of their job in previous research [30, 32, 33].

### Study design and sample

The study used data collected within the context of the impact evaluation of the Results-based Financing for Maternal and Newborn Care (RBF4MNH) Initiative, implemented in the country between 2013 and 2018. The impact evaluation covered 28 primary-level and five secondary-level health facilities providing emergency obstetric care across the four study districts (eight or nine facilities per district). The selection of intervention and comparison health facilities is described in detail elsewhere [34]. Data was collected from all 33 facilities just before (March/April 2013) and approximately 2 years (June/July 2015) after the start of RBF4MNH. For the purpose of this study, we pooled the 2013 and 2015 data. The role of RBF4MNH is not the focus in this study, but we controlled for time of data collection and RBF4MNH exposure (i.e., working in an RBF4MNH facility) in all analyses.

At health worker level, in all 33 study facilities, a repeated cross-sectional survey was performed in 2013 and 2015. Data were collected using a structured survey, administered face-to-face by trained interviewers with the support of tablet computers, in English which is the working language in Malawi.

All health workers providing maternal health care services (i.e., clinical officers, medical assistants, registered/enrolled nurse/midwives, nurse-midwife technicians) who had worked at the health facility for at least 3 months and who were available at the time of data collection were sampled. In total, 174 health workers were interviewed, 74 in 2013 and 100 at 2015. Due to frequent turnover of staff in the Malawian setting and the rotational nature of service organization, only 10% of health workers were interviewed both in 2013 and 2015. Table 1 provides an overview over the sample and key demographic characteristics.

**Table 1:** Sample characteristics

	Number	Percentage
Total	174	100
Sex		
Female	106	60.9
Male	68	39.1
Health worker type		
Clinical officer/medical assistant	31	17.8
Nurse	143	82.2
Health facility in-charge		
Yes	23	13.2
No	151	86.8
Level of care		
Primary	109	62.6
Secondary	65	37.4
Data collection year		
2013	74	42.5
2015	100	575
RBF4MNH exposure (i.e., working in an intervention facility)		
Yes	74	42.5
No	100	57.5
	mean	sd
Years at current health facility	4.3	5.6
Years in health care service	10.3	12.1

### Variables and their measurement

#### Outcome variable

Psychological wellbeing of health workers was measured using the WHO-5 Wellbeing Index (abbreviated as “WHO-5” in the following), a short, disease-unspecific, and non-invasive self-rating scale [35, 36] (see Table 2). The WHO-5 has been translated into over 30 languages and used vastly in a wide range of fields of application, although with health workers in a LLMIC only in the study in Zimbabwe mentioned earlier, where it was not validated [22]. Despite this lack of context-specific validation studies, we have no reason for serious doubts in its cross-cultural validity due to the straightforward language and item wording which does not appear to be particularly sensitive to cultural norms [36]. Both Cronbach’s *α* (.72) and factor analysis results (Loevinger *H* = .380, *p* = 0.000) support the notion that the WHO-5 items measure a unidimensional wellbeing factor.

**Table 2:** WHO-5 Wellbeing Index [35]

Please indicate for each of the five statements which is closest to how you have been feeling over the last 2 weeks.				
Over the past 2 weeks…	Most of the time	More than half of the time	Less than half of the time	Never
… I have felt cheerful and in good spirits	3	2	1	0
… I have felt calm and relaxed	3	2	1	0
… I have felt active and vigorous	3	2	1	0
… I woke up feeling fresh and rested	3	2	1	0
… my daily life has been filled with things that interest me	3	2	1	0

Scoring: The raw score is calculated by summing the points associated with the answers to the five statements. The raw score therefore ranges from 0 to 15, 0 representing the worst possible and 15 the best possible wellbeing. For the analyses, the raw score was linear transformed to decimal values between 0 and 1, corresponding to percentage of maximum score

A number of studies primarily in high-income settings have further shown the usefulness, validity, and sensitivity of the WHO-5 as a screening tool for mental illness. Based on this research, WHO-5 scores below 50% of the maximum score (i.e., below 8 on the 0–15 range) are considered indicative of potentially clinically relevant mental health problems. If the WHO-5 is used as a mental health screening tool, it is recommended that individuals scoring below this threshold undergo more intensive testing for mental illness [36]. We are not aware of any studies investigating the validity of this threshold in LLMIC generally or in sub-Saharan Africa more specifically.

We used the WHO-5 both in continuous form—to reflect our main conceptualization of PW as a continuum—and in dichotomized form along the 50% threshold to determine the proportion of the sample with WHO-5 scores indicative of potentially clinically relevant poor PW. To address the issue of lack of context-specific validation of the 50% threshold, we performed additional sensitivity analyses moving the threshold to (approximately) 40% (below 6 on the 0–15 range) and 60% (below 10).

#### Explanatory variables

Table 3 provides an overview of potential individual-level characteristics associated with PW, as well as details on measurement for non-standard variables. The choice of variables resulted from joint consideration of the conceptual framework presented in the introduction, and availability of respective variables in the questionnaire.

**Table 3:** Explanatory variables and their measurement

Variable	Measurement	Distribution	
Basic demographic and other characteristics			
Sex (male, female)		See Table 1	
Number of years in health care service			
Responsibility as health facility in-charge (yes, no)			
Cadre (clinical officer/medical assistant, nurse)			
Level of care (primary, secondary)			
Data collection year (2013, 2015)			
Exposure to the RBF4MNH Initiative (yes, no)			
Relationship		Yes No	44.8% 55.2%
Children and other dependents		Yes No	77.0% 23.0%
Clinical competence			
Any professional training within the last year		Yes No	57.5% 42.5%
Clinical knowledge	Clinical case vignettes pertaining to maternal care; see for details [37]	Mean sd	0.59 0.24
Perceived competence*	“I am self-assured about my capabilities to perform my work activities.”	Mean sd	0.86 0.12
Organizational support			
Any supervision within the last month		Yes No	58.1% 41.9%
Perceived supervisor support*	4 Likert items, e.g., “My supervisor takes pride in my accomplishments at work.”	Mean sd	0.64 0.18
Perceived co-worker support & team work*	8 Likert items, e.g., “The people I work with encourage each other to work together.”	Mean sd	0.75 0.14
Satisfaction with working conditions			
Satisfaction with physical work environment*	“How satisfied are you with …” 1) availability of medicine, 2) availability and condition of equipment, 3) availability of other supplies, 4) infrastructure	Mean sd	0.48 0.29
Satisfaction with remuneration*	“How satisfied are you with …” 1) salary, 2) benefits (incl. housing, allowances, bonuses)	Mean sd	0.18 0.27
Satisfaction with demands of the job*	“How satisfied are you with …” 1) variety and challenges at work, 2) demands of the job, 3) workload	Mean sd	0.50 0.27
Satisfaction with interpersonal relationships at work*	“How satisfied are you with your working relationship with …” 1) coworker, 2) district/MoH staff, 3) management staff, 4) traditional leaders, 5) community health workers, 6) community members	Mean sd	0.72 0.17
Motivation			
Intrinsic motivation*	3 Likert items, e.g., “I work in this job because my work has become a fundamental part of who I am.”	Mean sd	0.77 0.14
Extrinsic motivation*	“I work in this job for the income it provides me.”	Mean sd	0.50 0.29

Note: Responses to Likert items (marked *) were given on a scale from 1 (strongly disagree/unsatisfied) to 5 (strongly agree/very satisfied). For variables measured with more than one Likert item, the unweighted mean of responses to all items was calculated. At the analytical level, all variables measured with multiple items were rescaled to range from 0 (lowest level) to 1 (highest level) for ease of interpretation

### Analysis

In a first step, we performed *χ*^2^ tests for subsample differences in PW on key variables. We then employed linear (continuous outcome) and logistic (dichotomous outcome) regression models with standard errors clustered at facility level to determine the strength of association of the individual-level factors in Table 3 with PW. Data were complete for the WHO-5. For the predictor variables, data were missing for less than 2% of the sample for all variables except age (3.5%) and were imputed using modes/means in the respective RBF4MNH impact evaluation study arm*data collection year subsample.

## Results

### Psychological wellbeing levels

**Fig. 2:**
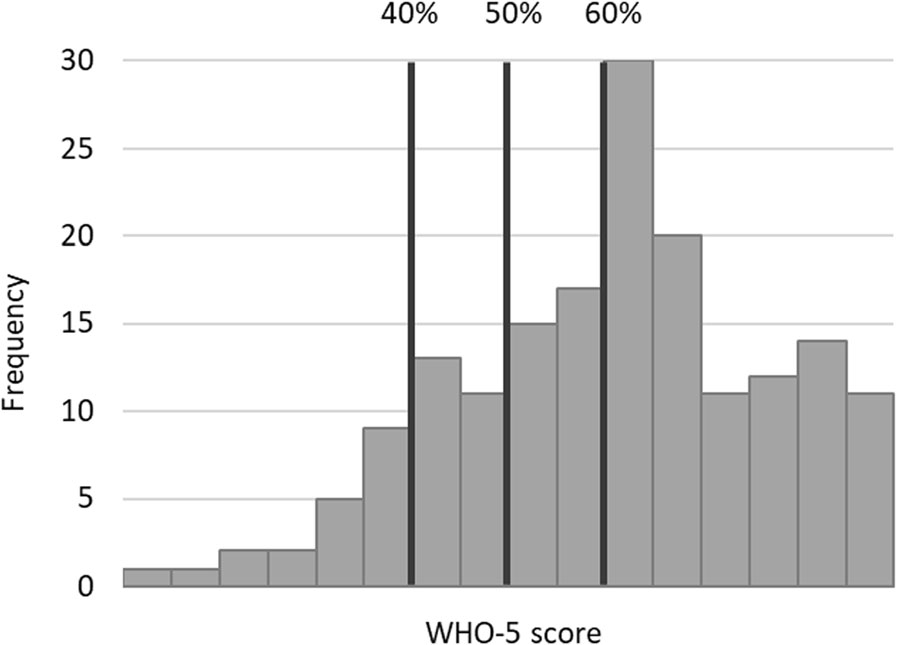
Distribution of WHO-5 scores among respondents. Note: vertical lines indicate the thresholds. WHO recommended that individuals having scores below the 50% threshold undergo in-depth mental health testing

Figure 2 shows the distribution of health workers’ scores on the WHO-5. The vertical lines indicate the 40%, 50%, and 60% thresholds, respectively. Scores below the 50% threshold are considered as indicators of clinically relevant mental health problems as explained above. In our sample, 25% of respondents scored below this threshold, 4% even below 25% of the maximum WHO-5 score. Twelve percent of respondents scored below 40% of the maximum, and 44% below the 60%. On the continuous WHO-5, respondents’ average score was at 64% of the maximum (sd = 22%).

Table 4 shows that there were substantial differences in PW by data collection year for the 50% and 40% thresholds, in that the proportion of health workers with poor PW levels was substantially lower in 2015 than in 2013, and by responsibility at the health facility for the 60% threshold, in that health facility in-charges indicated poorer wellbeing. No significant differences were found for sex and cadre.

**Table 4:** Proportion of health workers with WHO-5 scores below the 50%, 40%, and 60% thresholds overall and by key demographic subgroups

	Proportion of sample with poor wellbeing (50% threshold)	Proportion of sample with poor wellbeing (40% threshold)	Proportion of sample with poor wellbeing (60% threshold)
Total	25.3%	11.5%	43.7%
Sex			
Female	23.6%	12.3%	41.5%
Male	27.9%	10.3%	47.1%
Health facility in-charge			
Yes	34.8%	13.0%	65.2%*
No	23.8%	11.3%	40.4%*
Health worker type			
Clinical officer/medical assistant	32.3%	9.7%	58.1%
Nurse	23.8%	11.9%	40.6%
Data collection year			
2013	35.1%*	18.9%*	48.7%
2015	18.0%*	6.0%*	40.4%

*Difference is statistically significant

### Factors associated with psychological wellbeing

Table 5 shows the results of the multivariate analysis to determine factors associated with PW. The first column gives results using the WHO-5 continuous score, representing a conceptualization of PW along a continuum from low to high. The other columns show results using the WHO-5 dichotomized score according to the 50% threshold recommended by WHO as well as the alternative 40% and 60% thresholds, representing a more clinical conceptualization of good versus poor PW, the latter potentially requiring treatment. Overall, the multivariate models were able to explain between 20% and 36% in variance in PW, depending on the model used. In the following, we briefly summarize the results, using “statistically significant” to refer to coefficients for which the 95% confidence interval does not include zero.

**Table 5:** Factors associated with psychological wellbeing

	Continuous WHO-5 (min 0, max 15)	Dichotomized WHO-5 (0 = poor, 1 = adequate wellbeing)		
		50%	40%	60%
	Coef. [95% CI]	Coef. [95% CI]	Coef. [95% CI]	Coef. [95% CI]
Sex (0 = female, 1 = male)	.02 [− .83, .88]	− .30 [− 1.11, .51]	− .37 [− 1.52, .78]	− .34 [− .94, .27]
Years in service	.00 [− .04, .05]	− .01 [− .05, .03]	− .06 [− .11, − .01]	− .03 [− .06, .01]
Health facility in-charge (0 = no, 1 = yes)	− 1.12 [− 2.68, .44]	− .69 [− 2.32, .95]	− 1.22 [− 4.48, 2.03]	− 1.32 [− 2.41, − .23]
Cadre (0 = clinical officer/medical assistant, 1 = nurse)	− .05 [− 1.65, 1.56]	− .32 [− 2.16, 1.52]	− 1.42 [− 4.88, 2.05]	− .42 [− 1.71, .87]
Relationship status (0 = not in relationship, 1 = in rel.)	− .49 [− 1.21, .24]	− .70 [− 1.22, − .18]	− .71 [− 1.97, .54]	− .24 [− .76, .29]
Children/other dependents (0 = none, 1 = one or more)	− .08 [− 1.39, 1.24]	.19 [− .85, 1.24]	.91 [− 1.38, 3.20]	.39 [− .42, 1.21]
Any prof. training in last year (0 = no, 1 = yes)	− .94 [− 1.71, − .17]	− .43 [− 1.29, .43]	− .56 [− 1.91, .81]	− .73 [− 1.39, − .08]
Clinical knowledge	− 1.23 [− 3.74, 1.28]	− 1.26 [− 3.43, .91]	− 2.35 [− 4.29, − .40]	− .11 [− 1.83, 1.61]
Perceived competence	3.58 [− .22, 7.38]	4.13 [− .44, 8.70]	1.12 [− 2.64, 4.88]	3.47 [1.95, 6.74]
Supervision in last month (0 = no, 1 = yes)	.50 [− .54, 1.54]	.29 [− .56, 1.14]	− .27 [− 1.32, .78]	.47 [− .22, 1.16]
Perceived supervisor support	.05 [− 2.85, 2.94]	1.70 [− .83, 4.23]	3.20 [.08, 6.32]	− .34 [− 2.72, 2.04]
Perceived co-worker support & team work	− .67 [− 4.46, 3.11]	− .02 [− 2.58, 2.53]	− .80 [− 6.05, 4.45]	.72 [− 2.20, 3.64]
Satisfaction with physical work environment	1.24 [− 1.21, 3.68]	.52 [− 1.79, 2.83]	− 1.59 [− 4.20, 1.01]	1.28 [− .36, 2.92]
Satisfaction with remuneration	− .67 [− 2.83, 1.48]	− .29 [− 2.08, 1.49]	.28 [− 4.25, 4.82]	− .45 [− 2.08, 1.18]
Satisfaction with job demands	1.70 [− .34, 3.74]	1.24 [− .62, 3.10]	1.57 [− .78, 3.93]	2.19 [.79, 3.59]
Satisfaction with interpersonal relationships at work	5.11 [1.89, 8.33]	2.03 [− .48, 4.55]	7.07 [3.01, 11.13]	2.42 [.06, 4.78]
Intrinsic motivation	1.51 [− 2.00, 5.02]	− .30 [− 3.62, 3.04]	.01 [− 6.02, 6.04]	1.24 [− .97, 3.44]
Extrinsic motivation	.06 [− 1.26, 1.37]	.30 [− 1.03, 1.63]	.46 [− 1.23, 2.14]	− .52 [− 1.54, .50]
Health facility level (0 = primary, 1 = secondary)	1.34 [.08, 2.60]	.40 [− .85, 1.65]	1.27 [− .07, 2.61]	.78 [− .27, 1.82]
Data collection year (0 = 2013, 1 = 2015)	.41 [− .40, 1.21]	.84 [− .23, 1.90]	8.89 [7.76, 10.03]	− .12 [− .61, .37]
RBF4MNH exposure (0 = no, 1 = yes)	− 1.03 [− 2.49, .43]	− 1.53 [− 3.40, .35]	− 16.65 [− 18.9, − 14.4]	− .08 [− .96, .80]
*Model statistics*	*F = 34.3* *p = .000* *R*^*2*^ *= .28*	*Wald χ*^*2*^ *= 110* *p = .000* *Pseudo R*^*2*^ *= .22*	*Wald χ*^*2*^ *= 749* *p = .000* *Pseudo R*^*2*^ *= .36*	*Wald χ*^*2*^ *= 113* *p = .000* *Pseudo R*^*2*^ *= .20*

#### Basic characteristics

Sex and whether the health worker had dependents to care for were not significantly associated with PW. Cadre was also not significantly associated with PW, but coefficients pointed in the direction of clinical officers experiencing higher PW than other health workers in all models. Health facility in-charges tended to have lower PW than health workers without management responsibility, although statistically significant only when using the WHO-5 dichotomized at the 60% threshold. Health workers who had been in service for longer tended to indicate lower PW, although statistically significant only when using the WHO-5 dichotomized at the 40% threshold. Finally, health workers in a relationship reported lower PW, although statistically significant only when dichotomizing the WHO-5 at the 50% threshold.

#### Clinical competence

Respondents having received training in the last year reported lower PW, although statistically significant so only when using the WHO-5 continuously or dichotomized at the 60% threshold. Respondents with higher levels of perceived own competence tended to report higher PW, although statistically significant only when using the WHO-5 dichotomized at the 60% threshold. Respondents with higher general clinical knowledge tended to report lower PW (statistically significant only for the WHO-5 dichotomized at the 40% threshold).

#### Organizational support

Organizational support factors were not significantly associated with PW when controlling for other factors, and there were no consistent patterns in (non-significant) coefficients either. This is with the exception of perceived supervisor support, which was positively related to PW when using the WHO-5 dichotomized at the 40% threshold.

#### Job satisfaction

Health workers with higher satisfaction with interpersonal relationships at work reported higher PW, statistically significant for all but the 50% threshold. Higher satisfaction with the demands of the job was also associated with higher PW, although statistically significant only when using the WHO-5 dichotomized at the 60% threshold. Satisfaction with the physical work environment and with remuneration, in contrast, were not associated with PW.

#### Motivation

Neither intrinsic nor extrinsic motivation was significantly related to PW.

#### RBF4MNH exposure

We found no association of RBF4MNH exposure with PW, except when dichotomizing the WHO-5 at the 40% threshold, in that respondents who had experienced RBF4MNH were more likely to be in the poor PW category.

#### Year of data collection

As indicated by the bivariate analyses, the multivariate analyses suggest that the proportion of health workers with poor PW levels was lower in 2015 as opposed to 2013, but this was statistically significant only when dichotomizing the WHO-5 along the 40% threshold.

#### Level of care

Finally, health workers working in secondary-level hospitals as opposed to primary-level health centers tended to have higher PW, but statistically significantly so only when using the continuous WHO-5.

## Discussion

In line with the minimal prior research on health workers’ psychological wellbeing reviewed in the introduction, our study shows concerning levels of poor PW. Approximately 25% of the study sample scored below 50% of the maximum score, below which WHO recommends more in-depth mental health screening [35, 36]. Approximately half of the participants had scores not deemed of clinical concern according to the WHO recommendation, but which were still far below maximum wellbeing scores. Only about one quarter of respondents indicated high wellbeing levels. Unfortunately, we were unable to assess consequences of poor and suboptimal PW in our study. However, beyond the obvious concern for individual health, the available HIC literature shows strong links between low PW and substandard work performance [8, 10–12], underlining the importance of the issue from a health system perspective.

Our study is one of the first to measure psychological wellbeing in a low-income country setting. An important limitation is that to our knowledge, there are no validation studies of the WHO-5 in Malawi or in other LLMIC to date. While we have little reason to doubt the WHO-5’s validity and usefulness when used as a continuous variable as explained in the “Methods” section, we cannot be certain that the measurements fully reflect health workers’ psychological wellbeing levels. Most importantly, it is unclear whether the threshold indicated by WHO to differentiate healthy from non-healthy states is valid for health workers in LLMIC. In our study, lowering the threshold from 50 to 40% of the maximum WHO-5 score resulted in a halving of the proportion of respondents classified as of poor PW, while lifting it to 60% resulted in almost a doubling of the proportion. This indicates an imperative need for validation research, linking the WHO-5 to other mental health measures and tangible outcome criteria. At the same time, even when lowering the threshold substantially, the remaining proportion of the sample classified as of poor wellbeing is fairly substantive and highly relevant from a health systems perspective.

In this context, it is also important to consider that the sample is not fully representative of the health worker population. Rather, only health workers present at the workplace were interviewed, thereby possibly excluding health workers unable to report to work due to particularly poor psychological wellbeing. Estimates of psychological wellbeing in our study, therefore, are likely positively biased. Further, the study only included health workers providing maternity care services, a particularly high-burden work environment characterized by high emergency load, and might not fully generalize to other health worker cadres. In future research, inclusion of both a broader spectrum of health workers as well as representative samples including also such health workers which are not readily available at the workplace would be of immense value.

A comparison of our finding to those of the two previous studies conducted in Malawi is difficult as the latter measured specifically burnout, characterized by emotional exhaustion, depersonalization, and reduced personal accomplishment, rather than generalized psychological wellbeing as we did. In 2009, in a sample comparable to ours, McAuliffe et al. [18] found high levels of burnout among 5–31% (depending on symptom) of respondents. Assuming that the WHO-5 measures a construct somewhat related to burnout, our findings indicate a similar situation five to seven years later. In contrast, Thorsen et al. [23], also in 2009, found much higher burnout levels (68%) among maternal care staff, which might however be explained by their study being limited to only one district hospital.

A direct comparison of our findings with PW among health workers in other LLMIC is also difficult due to differing measures. To our knowledge, the only study having also used the WHO-5 was conducted in Zimbabwe [22]. Average sample scores ranged from 80 to 88% of the maximum, depending on the data collection time point and subsample, and are therefore substantially higher than the average 64% of the maximum in our Malawi study.

Analyses of factors associated with wellbeing allow only few tangible conclusions as factors significantly associated with PW varied by how the WHO-5 was used (continuous vs. categorical; threshold). Coefficients carried the same sign across models for only about half of the included variables, but for the majority of variables reached statistical significance for none or only one out of the four models. Contrary to our expectations, coefficients for sex, cadre, having children or other dependents, supervision, perceived coworker support and team work, satisfaction with the physical work environment, satisfaction with remuneration, and intrinsic and extrinsic motivation were consistently not statistically significant. Only for two variables did we find somewhat consistent significant associations: First, satisfaction with interpersonal relationships at work was positively associated with PW in three out of four models, underlining the importance of social relationships evidenced in studies from other settings [38, 39]. Second, whether the respondent had received any professional training in the last year was negatively associated with PW. One possible explanation is that health workers having recently received training might be more aware of their suboptimal working conditions which make the provision of high-quality services very difficult, negatively weighing on their PW. Possible alternative explanations include poor training quality, or factors concurrently associated with PW and actively seeking out training, such as anxiety. This might be a valuable area for future exploration. Particularly with respect to potential interventions to improve health worker PW, our findings imply that strengthening health workers’ clinical skills alone might not be effective in a severely resource-limited setting where clinical skills cannot always be readily translated into practice.

Our study is limited in that it relies on cross-sectional data and is therefore unable to identify causal relationships, which should be kept in mind when interpreting the findings. Limited to four districts and a sample size of 174, the study is clearly not able to close the existing knowledge gap, but is rather intended as one contribution towards an evidence base in need of further expansion. Further, the study used data collected for a different primary purpose, and questionnaires did not include all variables potentially relevant to PW. Results show that the included variables explain only between 20 and 36% of variance in PW, indicating the importance of other work-related and non-work-related factors at the individual and higher organizational levels in determining psychological wellbeing. More comprehensive, focused research would therefore be highly desirable for a more comprehensive picture of determinants of wellbeing of health workers.

One final aspect to underline again is that findings regarding factors associated with PW differ somewhat depending on how the WHO-5 is used. Coefficients on key variables tend to be aligned in direction, but not necessarily in magnitude, and often emerge as significantly different from zero only in one or some analyses, but not in others. Beyond a need for further research to confirm emerging findings, this underlines the above-discussed need for validation research in order to be able to make fully meaningful and valuable use of the WHO-5 as a screening tool applicable in HRH practice.

## Conclusion

The high proportion of health workers with poor wellbeing scores is concerning in light of strong links between wellbeing and work performance, particularly in a heavily constrained HRH situation such as in Malawi. While more research is needed to draw conclusions and provide detailed recommendations as to how to enhance wellbeing, our results underline the importance of including the issue in the human resources for health discourse and research agenda.

## Data Availability

The datasets used and/or analyzed during the current study are available from the corresponding author on reasonable request.

## Abbreviations

DHMT: District Health Management Team
HIC: High-income country
HRH: Human resources for health
LLMIC: Low- and lower-middle-income country
PW: Psychological wellbeing
RBF4MNH: Results-based Financing for Maternal and Newborn Health
WHO: World Health Organization
WHO-5: WHO-5 Wellbeing Index

## References

[1] World Health Organization (2007). Everybody’s business: strengthening health systems to improve health outcomes. WHO’s framework for action.

[2] Campbell J, Dussault G, Buchan J, Pozo-Martin F, Guerra Arias M, Leone C, Siyam A, Cometto G (2013). A universal truth: no health without a workforce. Forum report, Third Global Forum on Human Resources for Health, Recife, Brazil.

[3] Jennings BM, Hughes RG (2008). Work stress and burnout among nurses: role of the work environment and working conditions. Patient safety and quality: an evidence-based handbook for nurses.

[4] Stansfeld SA, Rasul FR, Head J, Singleton N (2011). Occupation and mental health in a national UK survey. Soc Psychiatry Psychiatr Epidemiol.

[5] European Union (2016). European framework for action on mental health and wellbeing.

[6] OECD (2012). Sick on the job? Myths and realities about mental health and work.

[7] World Health Organization (2010). Healthy workplaces: a model for action: for employers, workers, policymakers and practitioners.

[8] Aiken LH, Sermeus W, Van den Heede K, Sloane DM, Busse R, McKee M (2012). Patient safety, satisfaction, and quality of hospital care: cross sectional surveys of nurses and patients in 12 countries in Europe and the United States. BMJ..

[9] McVicar A (2003). Workplace stress in nursing: a literature review. J Adv Nurs.

[10] Hall LH, Johnson J, Watt I, Tsipa A, O’Connor DB (2016). Healthcare staff wellbeing, burnout, and patient safety: a systematic review. PLoS One.

[11] Wilkinson H, Whittington R, Perry L, Earnes C (2017). Examining the relationship between burnout and empathy in healthcare professionals: a systematic review. Burn Res.

[12] Davey MM, Cummings G, Newburn-Cook CV, Lo EA (2009). Predictors of nurse absenteeism in hospitals: a systematic review. J Nurs Manag.

[13] Dugani S, Afari H, Hirschhorn LR, Ratcliffe H, Veillard J, Martin G (2018). Prevalence and factors associated with burnout among frontline primary health care providers in low- and middle-income countries: a systematic review. Gates Open Res.

[14] Ahmad W, Taggart F, Shafique MS, Muzafar Y, Abidi S, Ghani N, Malik Z, Zahid T, Waqas A, Ghaffar N (2015). Diet, exercise and mental-wellbeing of healthcare professionals (doctors, dentists and nurses) in Pakistan. PeerJ..

[15] Bonenberger M, Aikins M, Akweongo P, Wyss K (2014). The effects of health worker motivation and job satisfaction on turnover intention in Ghana: a cross-sectional study. Hum Resour Health.

[16] Dieleman M, Biemba G, Mphuka S, Sichinga-Sichali K, Sissolak D, van der Kwaak A, van der Wilt GJ (2007). ‘We are also dying like any other people, we are also people’: perceptions of the impact of HIV/AIDS on health workers in two districts in Zambia. Health Policy Plan.

[17] Mbindyo PM, Blaauw D, Gilson L, English M (2009). Developing a tool to measure health worker motivation in district hospitals in Kenya. Hum Resour Health.

[18] McAuliffe E, Bowie C, Manafa O, Maseko F, MacLachlan M, Hevey D (2009). Measuring and managing the work environment of the mid-level provider -- the neglected human resource. Hum Resour Health.

[19] Muliira RS, Ssendikadiwa VB (2016). Professional quality of life and associated factors among Ugandan midwives working in Mubende and Mityana rural districts. Matern Child Health J.

[20] Mutale W, Ayles H, Bond V, Mwanamwenge M, Balabanova D (2013). Measuring health workers’ motivation in rural health facilities: baseline results from three study districts in Zambia. Hum Resour Health.

[21] Nantsupawat A, Nantsupawat R, Kunaviktikul W, Turale S, Poghosyan L (2016). Nurse burnout, nurse-reported quality of care, and patient outcomes in Thai hospitals. J Nurs Scholarsh.

[22] Nguyen HTH, Gopalan S, Mutasa R, Friedman J, Das AK, Sisimayi C (2015). Impact of results-based financing on health worker satisfaction and motivation in Zimbabwe.

[23] Thorsen VC, Teten Tharp AL, Meguid T (2011). High rates of burnout among maternal health staff at a referral hospital in Malawi: a cross-sectional study. BMC Nurs.

[24] Mills A (2014). Health care systems in low- and middle-income countries. N Engl J Med.

[25] World Health Organization (2013). Mental health action plan 2013–2020.

[26] Maslach C, Schaufeli WB, Leiter MP (2001). Job burnout. Annu Rev Psychol.

[27] National Statistical Office, ICF International (2016). Malawi Demographic and Health Survey 2015–16.

[28] Ministry of Health Malawi, ICF International (2014). Malawi Service Provision Assessment (SPA) 2013–14.

[29] Bradley S, Kamwendo F, Chipeta E, Chimwaza W, de Pinho H, McAuliffe E (2015). Too few staff, too many patients: a qualitative study of the impact on obstetric care providers and on quality of care in Malawi. BMC Pregnancy Childbirth.

[30] Manafa O, McAuliffe E, Maseko F, Bowie C, MacLachlan M, Normand C (2009). Retention of health workers in Malawi: perspectives of health workers and district management. Hum Resour Health.

[31] Chimwaza W, Chipeta E, Ngwira A, Kamwendo F, Taulo F, Bradley S, McAuliffe E (2014). What makes staff consider leaving the health service in Malawi?. Hum Resour Health.

[32] Goldberg AB, Ron LI (2012). Understanding the complex drivers of intrinsic motivation for health workers in Malawi. Health Systems 20/20 project report.

[33] Lohmann J, Muula AS, Houlfort N, De Allegri M (2018). How does performance-based financing affect health workers’ intrinsic motivation? A self-determination theory-based mixed-methods study in Malawi. Soc Sci Med.

[34] Brenner S, Muula AS, Robyn PJ, Bärnighausen T, Sarker M, Mathanga DP (2014). Design of an impact evaluation using a mixed methods model – an explanatory assessment of the effects of results-based financing mechanisms on maternal healthcare services in Malawi. BMC Health Serv Res.

[35] World Health Organization (1998). Wellbeing measures in primary health care. The Depcare project.

[36] Topp CW, Østergaard SD, Søndergaard S, Bech P (2015). The WHO-5 Well-Being Index: a systematic review of the literature. Psychother Psychosom.

[37] Wilhelm D, Lohmann J, De Allegri M, Chinkhumba J, Muula AS, Brenner S. (2019). Quality of maternal obstetric and neonatal care in low-income countries: development of a composite index. BMC Med Res Methodol, 19:154. 10.1186/s12874-019-0790-0 PMC6637560 31315575

[38] Kawachi I, Berkman LF (2001). Social ties and mental health. J Urban Health.

[39] Stansfeld S, Candy B (2006). Psychosocial work environment and mental health--a meta-analytic review. Scand J Work Environ Health.

